# Community perceptions of unintentional child injuries in Makwanpur district of Nepal: a qualitative study

**DOI:** 10.1186/1471-2458-14-476

**Published:** 2014-05-20

**Authors:** Puspa Raj Pant, Elizabeth Towner, Paul Pilkington, Matthew Ellis, Dharma Manandhar

**Affiliations:** 1 Centre for Child and Adolescent Health, University of the West of England, Bristol, UK; 2 School of Social and Community Medicine, University of Bristol, Bristol, UK; 3 Mother and Infant Research Activities (MIRA), Kathmandu, Nepal

**Keywords:** Child injury prevention, Qualitative, Child injuries, Nepal, Low income countries

## Abstract

**Background:**

In Nepal, childhood unintentional injury is an emerging public health problem but it has not been prioritised on national health agenda. There is lack of literature on community perceptions about child injuries. This study has explored community perceptions about child injuries and how injuries can be prevented.

**Methods:**

Focus group discussions were conducted with mothers, school students and community health volunteers from urban and rural parts of Makwanpur district in Nepal. FGDs were conducted in Nepali languages. These were recorded, transcribed and translated into English. A theoretical framework was identified and thematic analysis conducted.

**Results:**

Three focus group discussions, with a total of 27 participants, took place. Participants were able to identify examples of child injuries which took place in their community but these generally related to fatal and severe injuries. Participants identified risk factors such as the child’s age, gender, behaviours and whether they had been supervised. Consequences of injuries such as physical and psychological effects, impact on household budgets and disturbance in household plans were identified. Suggestions were made about culturally appropriate prevention measures, and included; suitable supervision arrangements, separation of hazards and teaching about safety to the parents and children.

**Conclusion:**

Community members in Nepal can provide useful information about childhood injuries and their prevention but this knowledge is not transferred into action. Understanding community perceptions about injuries and their prevention can contribute to the development of preventive interventions in low income settings.

## Background

Childhood unintentional injuries are a leading cause of death globally among children and young people aged 0–17 years [1]. Ninety-five per cent of childhood injury deaths occur in low- and middle- income countries (LMICs) [2]. During the year 2010, about 3,400 Nepalese children and adolescents (<20 years) died from unintentional injuries [3]. In the year 2010, injuries claimed 1,900 deaths, 13% of the child deaths which occurred between the age of 1 to 59 months [4]. Injuries were the second leading cause of death among children aged 1 to 59 months after diarrhoea. The latest updates on the Global Burden of Diseases shows that injury is the fourth leading cause of deaths among children below 15 years of age. Therefore, urgent action for comprehensive information on the type, causes and risk factors of childhood injuries, along with their socio-economic impact on individuals and families is needed [5,6].

Injuries are associated with day-to-day activities and environments, which differ from place to place. To perceive something as risk or not risk for an injury is solely depends upon an individual’s understanding; which in turn attributed to perceived meaning, anticipated consequences and consideration of preventive and safety measures.

There is a growing literature of qualitative studies relating to unintentional injuries [7]. These include the dimensions and meaning of child supervision [8], preventability of injuries at home [9], parent’s knowledge, attitude and beliefs related to child injuries [10], knowledge and beliefs of young mothers [11]. These studies have explored a range of findings elucidating their association with the occurrence of an injury to children and preventability. However there is shortage of injury research literature from low- and middle- income countries. The number of hospital-based studies is relatively higher than community based studies; there are only a few qualitative studies are available from South-East Asia region. In Bangladesh, Mashreky [12] studied perceptions of rural people about childhood burns and Rahman [13] studied community perception of childhood drowning in rural Bangladesh. Both of these studies also explored opinions on injury prevention. In India, Jagnoor [14] studied elderly people’s perceptions about falls. Qualitative studies explore broader information on injuries, their consequences and prevention [15,16]. Qualitative findings help conceptualise the risk and provide with the hands-on possibilities of injury prevention [17].

Nepal has a population of about 27 million; about 45% percent of which comprise children and young people below the age of 18 years [18]. One-third of Nepal’s population lives on a daily income of less than 1 US$ [19]. The country is changing rapidly: its rate of urbanization is 5% per year [19]. In Nepal, 17% of its population lives in urban areas, where the population density is 9 times higher (1381/Km^2^) than rural areas (153/km^2^) [20]. Nepal has many factors that can contribute to its increased risk of injuries. Children in particular are exposed to multiple risk factors for injuries as 40% of the children aged 5–17 years are involved in labour and supporting domestic activities [21,22], in addition to living and working in challenging geographical settings. There is an urgent need to focus on childhood injuries and their prevention. There is no formal injury surveillance system in place in Nepal. In this context, qualitative investigations become highly significant because it provides a wide range of information about injuries in the communities.

Many people believe that injuries to be the result of ill fate, which prevents efforts to prevent injuries, if any. For example in Bangladesh, child drowning was believed to be 'natural and inevitable' and hence cannot be prevented [13]. The vulnerability of rural Nepalese children for injuries typically increases from the age of five years, as they begin to be more independent and separate from parental supervision, either looking after their younger siblings or helping in other domestic chores [22]. This study aimed to investigate perceptions of community members about the magnitude, risk factors, causes and consequences of injury; to describe their opinions about injury prevention; and to explore their own suggestions about injury prevention. This knowledge may help concerned authorities to take into account the expectations, perceptions and needs of the population for designing and implementing policies, messages and activities.

## Methods

Focus group discussions (FGDs) were conducted in three different groups: mothers, school students and community health volunteers in January 2011. Two focus groups were conducted in rural areas, Chhatiwan and Hatiya Village Development Committees (VDCs), and one in Hetauda municipality, an urban setting.

A semi-structured checklist (topic guide) was developed based on the available literature and was adapted from themes outlined in the Bangladesh Health and Injury Survey [23]. The topic guide sought the participants’ experience of childhood injuries in their communities and explored their understanding of the nature, causes, consequences, and treatment practices, as well as the participants’ opinions about the prevention of injuries.

Mothers, school students and female community health volunteers (FCHVs) were selected in separate groups for the FGDs since these people are supposedly the most affected and provide the intended information for this study. The selection of the groups was done according to convenience. Due to limitations of time and resources, separate groups for out of school children and fathers were not explored. However, FCHVs provided information about community, in general.

The focus group participants in Chhatiwan and Hatiya were recruited with the help of field staff of the MIRA project; Mother and Infant Research Activities (MIRA) is working in Makwanpur district since 1994. The school students were recruited by the author (PRP) with the help of the Head teacher. The aims and objectives of the FDGs were explained and group verbal consent obtained from the participants. The study guaranteed confidentiality of information and anonymity of participants. Ethical approval for conducting these studies was obtained from the Nepal Health Research Council beforehand.

Three members of the MIRA project, experienced in conducting and facilitating focus groups assisted the researcher (PRP) with note taking during each FGD. The note takers acted as ‘observers’ and took notes in which were later transcribed. Immediately following each focus group, the notes were discussed between the researcher and note taker to identify agreement on key issues.

Focus group discussions were recorded in full, with the permission of the participants. All the FGDs were conducted in the Nepali language. Express Dictate Digital Dictation Software (Free) of the NHC Software Inc. (http://www.nch.com.au/scribe/index.html) was used to transcribe verbatim the original transcripts into the full length Nepali language. Cassette tape data was transcribed using a cassette player. The transcription was conducted by the researcher (PRP). Once transcribed in Nepali, the scripts were translated into English by the researcher, in an attempt to retain the real meaning of the original statements of the speakers. The translated transcripts were read by a second researcher (ET) and minor edits were made. Basic data analysis was done using the qualitative data analysis software NVivo9.0 i.e. theme generation and compilation only. Themes were generated by reading the transcripts and relevant quotations organised accordingly.

## Results

The three focus group discussions involved 27 people, in total. The discussions lasted from 45 to 75 minutes. The health volunteers’ group included 8 females, aged 21 to 51 years, with four aged <40 years and four >40 years. All worked in different wards of Hatiya VDC in Makwanpur district where their work involved assisting in the government’s health awareness raising and mass treatment campaigns. The mothers’ group consisted of women from the *Majhi* (Fishermen) community (an indigenous group) in Chhatiwan VDC of Makwanpur district. All were aged <40 years, had at least one child and did not do paid work. Mothers only were chosen for FGDs because of two reasons: 1) male adults in rural communities of Nepal are absent from home for works elsewhere, and 2) female voice may be dominated if discussed in presence of their husbands. The students group was selected from a government High School in Hetauda. There were 10 participants, 6 males and 4 females. The students were aged 11–16 years and were studying in grades 6 to 9.

The findings are presented thematically according to the nine major themes which emerged from the discussion. The framework of the major themes and sub-themes is presented in Table 1.

**Table 1:** Schematic presentation of the major themes and topics discussed

**Themes**	**Area under discussion**
1. Beliefs about child injuries	• General perceptions
	• Knowledge about child injuries
	• Narration of different injury scenarios
	• Emotional and mythological perceptions
	• Sensitive issues
2. Reasons for child injuries	• How and why injuries occur
	• Vulnerable behaviours
	• Parents’ negligence
	• Risky environment
	• Lifestyle and circumstances
3. Risk groups for injuries	• Who are the most at risk?
	• Different population groups
	• Lack of supervision
4. Common places of injuries	• Locations identified where injuries occur
5. Treatment of injuries and health seeking behaviour	• Participants’ own experiences
	• Accessibility and affordability issues
	• Practices of care of the injured person
6. Consequences of child injuries	• Physical (visible) consequences
	• Emotional consequences
	• Added burden on the family
	• Economic burden
	• Lifelong disability
7. Perceptions about child injury prevention	• A difficult matter
	• Recognising the problem of child injuries
8. Who is responsible for child injury prevention	• Specific examples relating to the family and community
	• Specific examples relating to the role of the state

### Beliefs about child injuries

The term ‘Injury’ in local context means an event with discernible and severe outcomes, therefore, the mild to moderate injuries usually ignored. One of the participants of the health volunteer’s focus group stated that children need to be exposed to ‘minor bumps and bruises’ in order to be stronger in the future. They opined that by not having such exposures, children can lack resistance to minor bodily harms in the future. The opinions of some of the mothers’ group were also similar. The students’ had the opinion that injuries are quite common because they see them at home, on the roads and at school.

*“It is usual to have small cut bruises [showing scar on their hands], these all were the marks of cut we had in the past [explained how she got those scars].”* (Mother #7) *“You get cuts when you are cutting grass! [We don’t think] it is not important to mention here.”* (Mother #9)

In the beginning, most of the focus group participants found it difficult to give examples of injuries; particularly, the mothers. They instead cited some other childhood illnesses which were relevant to injury. Such expressions of the participants can also reflect the fact that they ignore child injuries in their daily lives. It was observed during the discussions that most of the participants initially provided examples of fatal or more noticeable incidents. They appeared to have seen numerous non-fatal incidents where children received injuries so they found it difficult to quote specific examples. A health volunteer described, as the very first example, an incident in which a sister became blinded with an arrow propelled by her brother while playing. Similarly, the participants of the mothers group described a tragic death of two children from a stationary tractor as the first example. A participant of the students’ group asked the facilitator whether it was relevant to discuss a fire related death of a girl which had took place in a dispute over dowry. Falls were the most frequently mentioned cause of non-fatal injuries to children by all focus group participants.

*Young children of age 4/5 years to 9/10 years at more risk of falls and fractures, also from falls from trees.* (FCHV #2)

*Children of age 5–10 years get more injuries from falls.* (FCHV #5)

*.... However, injuries below 5 years do occur at home; around home. One may fall while playing or walking and cries.* (Mother #9)

The participants of the health volunteers’ group provided detailed commentaries on several incidents of injuries (fatal and non-fatal) to children in their communities. The most common examples were related to falls, fractures, cuts and bruises, fire/burns, poisoning and motorcycle crashes. The mothers in the focus group were of the opinion that when children are at a risky place without supervision, fatal consequences may arise. The mothers stressed the need for supervision in a risky environment, giving an example of a girl of grade 6 or 7 who was drowned in a local stream.

*“Sometimes ago, another child was swimming in the same stream. She was studying in grade 6 or 7. She went (to swim) in the stream and never came back. I heard they went together with friends. Actually, (what happened) they started to carry one another and suddenly slipped on the rock. I think she must have fallen on the rocks. I don’t know if there was a big channel or what, they could not get out. Later when a man saw her body she was already died.”* (Mother #2)

The statement of a participant of the mothers’ focus group illustrates the lay perception about injuries. It was the case of her son who had a fall from a height for a second time at her own home while she was away working in the field. She thanked God and said ‘nothing’ had happened. It is important to note that a height of about 7 feet is sufficient to have severe injury for a child.

*“He was three years old...... He again fell off from upstairs in the same place. That time, I think, he landed on timber. We took him to Hetauda (hospital) straight away thinking that his backbone was broken, thank god nothing had happened (to his backbone). Actually, he had injured some bone, but we thought his backbone was broken.”* (Mother #8)

There was a mix of such perceptions behind an injury including simple bad coincidence, bad luck, witchcraft, ill-fate and the preventability of injuries. All the mothers’ group with one voice felt that *“It [injury] is said to happen because of an ill-fate. They are supposed to be unfortunate [children].”*

Some of the mothers believed that injuries occur due to the curses of other persons [witchcraft]. This also applies to other types of illnesses to children in many Nepalese traditional societies. A participant of the mothers’ group described a fatal injury which occurred to a boy who went to the flour-mill after his grandmother cursed him and wished him dead.

*…early in the morning his grandma cursed him – “may this (boy) die in the mill. What she said happened. He died. The poor boy was pulled in by the belt [of the machine].”* (Mother #6)

### Reasons for child injuries

Health volunteers put forward a range of reasons for injuries to children that they observed in the community during their work as FCHVs. They related the reasons for child injuries to variations in child care and supervision by parents at home. They also related children’s age as a responsible factor where there are different developmental stages. However, the mothers’ group perspectives about the causes of injuries to children were more specific. They felt that they lacked time to supervise their children because of other domestic activities, while the students’ opinion was that children’s own activities were also the causes of injuries; they illustrated this with examples of teenagers’ risky behaviours. A student participant told that injury may also occur completely inadvertently.

*“Only a few days ago, after 6th bell (in the afternoon), a boy rushed towards me. His eye was accidentally hit with my hand, but he accused me of causing the injury. [Actually it was him who rushed towards me!]”* (Grade 7 male student)

During all the three focus group, the researcher observed that the participants either blamed children or adults as the cause of injuries. They rarely suggested causes related to the structure of the places and children’s environments such as in the home, at school or on the roads that might be the main reason for injuries. Participants were found to be focussed more on risky activities and behaviours.

From the discussion with the health volunteers, the belief emerged that those children around 10 years of age start mimicking what they see around them or on cinema or television. They described an incident in which a child fractured his hand while jumping from the tree as he was acting like a movie hero.

*“Such bad incidents occur more if animals are slaughtered in front of children [of 3–5 years]. Once it occurred in my own family … children had seen a goat being slaughtered on the occasion of Dashain [festival]. After a few days, between Dashain and Tihar* (major Nepalese festivals)*, I saw the bigger one [4 year old boy] brought a knife [khurpa] and asked his younger sister to bow in the same position as the goat in order to behead her. I was there in the meantime [otherwise]…”* (health volunteer #3)

The school students identified the increasing use of motorcycles as the major reason for injuries among teenage children; they were focussing more on motorcycle crashes. When they were asked why they mentioned road traffic injuries so much, they replied that road crashes are on the increase in Nepal nowadays and that they were related to alcohol consumption by the driver. The students’ focus group believed that many (road) crashes occur because of alcohol and they strictly opposed driving under the influence of alcohol. In Nepal, the sale and consumption of liquor involving a minor (below 16 years) is prohibited by law [24] but due to the country's cultural diversity and home-brewing practice, this law has little influence. Similarly the legal age from driving in Nepal is set to be over 18 years [25].

The opinions of the health volunteers and students were very similar in relation to the use of motorcycles by teenagers and their risks of injuries. A health volunteer, who was also the grandmother of a teenage boy, described how her grandson behaviours led to injuries when he was riding a motorcycle without learning to ride it properly.

*“In spite of his uncle and father’s prohibition of using a motorcycle since he doesn’t know how to ride it properly he took it without listening to them. After some time he ploughed the motorbike into the field near Chundada! (all participants laugh on this statement). Then he returned and was lying down on the bed. We asked him what happened to him but he didn’t tell anything. When we checked his body we found he had bruises and wounds everywhere on his body.”* (health volunteer #7)

In a typical example, one of the health volunteers’ focus group mentioned an example of how her daughter incurred leg injuries while being transported on a bicycle to receive treatment for her hand injury.

*“She was climbing the stairs; suddenly she fell down and broke her hand. It was a Nepal bandh (strike) day. Her father was taking her to the hospital on a bicycle [since there were no vehicles operating due to the strike], [on the way] her leg was trapped into the wheels. Both hand and legs were broken.”* (health volunteer #6)

This example indicates clearly about lack of awareness about safety measures both before and after an injury. They did not have the idea that carrying an injured person on a bicycle is very risky and likely to worsen the condition of the patient. It is to note that the incident occurred on a strike day and there was no vehicular movement. In Nepal, access to health services from rural villages is not easy.

### High risk groups for injuries

Opinions of the participants of the focus groups were sought about who were the most at risk of injuries in the community. The groups of students and health volunteers had similar views that toddlers and teenagers are at the highest risk of injuries. The mothers were more focussed on the younger children less than 5/6 years of age and their supervision. The health volunteers’ group said that children aged 1–5 years were often wandering around randomly and exploring and that they needed to be supervised at all times. However this was not possible in their families. An example in which a 3–4 year old girl was burned by a rice-husk fire in the backyard while her mother was busy weaving carpets inside the home was provided to illustrate the situation.

The participants were asked to discuss: *who are at high risk of accidental injuries?* The health volunteers’ group provided a range of opinions. According to them people of the following groups are in the highest risk of having different type of injuries:

•males

•toddlers and children below 5 years of age

•children from 5 to 10 years of age

•children and young people from 10–22 years

•older people

•children from families with many children

•children from poor families

•children without proper supervision

The participants also felt that injuries can happen to anyone, any time at any place. But the members of the mothers’ group thought that children in rural villages suffer more from injuries than their urban counterparts.

*“In my opinion, there must be fewer incidents of injuries in towns than in the villages. In the town people do not have to go out of their rooms, most of the time. Here (in the village, we) have to go all around, neighbourhoods for any reasons or (to) work. They just watch TV indoors, in the town.”* (Mother #5)

### Common places of injuries

When participants provided real-life examples, which they had seen or experienced, they were able to describe the situation and places where those injuries occurred. However, the common places for injuries as discussed during the focus groups were home, farms and jungle, road/trails, stream/river, play areas and school.

Some of the activities included children picking fruit, or collecting firewood or fodder/leaves from trees, training/herding goats or cattle, all of which made children vulnerable to injuries. All these activities of children were often overlooked by the participants during the discussion, as they were prevalent as normal activities in a typical Nepali family. The mothers’ group participants observed that injuries are by-products of activities. Since such activities are very common occurrences injuries are also usual phenomena. Some of the students felt that injuries were also high in towns because of the increasing number of motorised vehicles and poor driving behaviour.

The common places and context of injuries are summarised in the Table 2.

**Table 2:** Common places of injury occurrence and activities

**Mechanism**	**Place**	**Activities leading to injuries**
Burns	Inside the home	Toddlers or small children walk into the hot ashes or cooking fire, playing with hot utensils around the kitchen, children roasting food (fish or potatoes) themselves.
Crush injuries	Work	Pulled by the flour-mill belts, crushed with rock or timber
Cut or sharp injuries	Farms/jungles	While collecting grass and firewood, while cutting grass or crops harvesting, while playing on the yard
Drowning	Water bodies	River, streams, water-bodies in unfamiliar places
Falls	Leisure	While playing in the playground, walking or wandering in streets/trails
	Public places	Accidental falls on steps of a temple, or in the farms, from ladders, steps,
	Leisure	Riding bicycles. Falls from the roof while flying kites
Poisoning	Indoor/Outdoor	Consume substances like ghee, poisons, ointment
	Indoors	Accidentally mixed with food stuffs kept indoor, consuming kerosene accidently.
RTIs	Roads	Pedestrians crossing the road, crushed by a vehicle, motor cycling
Snake bites	Outdoors	While walking, playing or working in the fields

### Treatment of injuries and health seeking

During the discussion, it was reported that the use of local contacts was practiced for treating injuries. For example, the mothers’ group said that they see the health volunteer (FCHV) in their community and she provides first aid and refer the child to the health facility. The health volunteers’ group said that it was common practice for people to delay seeking medical treatment for injuries. They also revealed that, people think the injury will heal after a time, and they only take the victim for treatment if the condition gets worse. Health volunteers told that, when they see an injured person, they generally encourage patients and say “don’t worry, it will be all right, you will recover”.

It appeared from the discussion that people tend to consult anyone they think could help. Since the health volunteers are involved in minor treatment they could be their first source of treatment or information. Health volunteers also complained that they were not equipped with adequate supplies so that they could not help people when needed. This may again force people to see the faith healers or apply home remedies. A number of common local treatments include the following:

#### Fracture

Splints are used to stabilise the fractured bones. Some herbal extracts are also applied. Injured persons are rushed to Health Centre after doing some first aid if it is head injury.

#### Burns

Use of Aloe Vera is very popular treatment of burns/scalds; running water is also used on burn or scalds. Application of eggs, raw tomato and potato to relieve burns was also reported by some participants. However, FCHVs generally ask people to use cold water in burns and rush to pharmacy or refer to the hospital etc.

#### Poisoning

Giving soap-water or dung-water as emetics was found to be practised for acute poisoning. Caustic soda-water is also given. They make the person forcefully swallow anything that cause vomiting.

#### Snakebite

Tourniquet is the most frequently applied means in case of a snake bite. It is used above the site of snakebite in order to prevent the poison from spreading and keep the person awake.

#### Cut wounds

Use herbal extract and a bandage in open cut wounds is the most prevalent in most of the rural areas. The bandage gauze may not be necessarily sterilised cotton; it can be any cloth or rag. People also apply crimson powder (red colour) and fine clay to stop bleeding from the wound. For severe laceration or chopped cases patients are rushed to the hospital/ health post, if possible.

#### First aid

The concept of doing first aid is based on the need of the situation, not as knowledge. The FCHVs do most of the first aid, so far, in the community.

### Consequences of injury

The participants of mother group very sadly recounted how a boy had become disabled after he had a fallen from height in his early childhood.

*“He was about one or one and half years old when he had a fall. [Now] he can’t read and write. This poor boy has a slow brain! [He can] recognise people, eat, and speak. He doesn’t know what to do, and how to do? [Pointing towards some basic tasks] For example: He defecates anywhere. I think such children are called mentally retarded.”* (Mother 2 and Mother #9)

They also described the situations in which the routine of their household chores is affected seriously.

*“Everything is messed up. Someone has to do it, who else would do it then? [It’s the mother].”* (Mother #9)

The participants of the health volunteers’ focus group provided examples of parents affected by psychological effects of their child death as a result of injuries.

*“The three children who had eaten the contaminated flatten rice (Chiura) in the morning before going to school, were taken to hospital by the teacher..... I think two of them died and he was the only one to survive. (No) two survived and only one died. The oldest one died. The mother is regularly taking antidepressants since she lost her son. She gets medicine from this health post.”* (Health volunteers #2 and #4)

All the focus group participants stated that to have an injured person in the family puts an additional burden on family dynamics. This situation may differ depending upon the socio-economic status of families. The magnitude of the burden of injury is determined by different factors. If the injured person is a breadwinner adult, there may be a severe financial crisis. If the injured person is a child, the parents or carers would have to be involved in his/her care and forsake other usual activities which may again bring economic and other hardships.

Some of the examples of non-economic impacts of injuries listed by the focus group participants are presented below:

family members required to look after the injured person, need extra time.

parents need to depend on other persons, carers or family members for patient-care while they are at work sometimes there is no option between usual household tasks and patient care.

parents or family members required to take days off from work to looking after the injured child.

#### Economic burden

The monetary costs to be borne by the family of an injured child were listed by the participants. These included the direct medical costs, accommodation and food for the attendee, borrowed loan amount or lost income, payments to carers.

*Oh its terrible, there are cows and buffaloes (cattle) kept at home, they are left to be starved (unattended).... It is very sad, disappointing. There is short of money for the treatment. [We] have to manage for other domestic chores. It [also] requires to look for money (borrow or loan) to take the injured person for treatments.* (Mothers #1, #6 and #9).

The students’ focus group stated that there are costs of employing another person for injured person’s care. There may be a reduction in family income or financial problems. They may have to take out loan to cover treatment costs, for those who do not have regular income or who are unemployed. They also emphasised that the situation is much worse when the injured person survives after severe injuries.

#### Lifelong disability

The mothers’ group participants cited examples of some 20 cases of child injuries, which included five disabling injuries and one child lost vision and other three were traumatised as a result of the injury. One of the mothers’ group participants gave an example of how a young girl had her leg amputated after it was crushed in a mill injury.

*“Yes, my cousin sister, she was also small at that time. ......... Her (grandma’s) shawl was caught by the belt of the mill and pulled her in. She escaped but the little girl’s leg was crushed into pieces. … she was taken … for treatment. That girl has now become 17–18 years. The injured leg had to be amputated and was replaced with duplicate leg using steel (prosthetic). That also cost the family dear.”* (Mother #8)

The health volunteers described how some parents did not take the injuries to their children seriously which result in complications. An example of how a child lost his vision due to delays in treatment was used as illustration.

*“The brother said; look at me sister now I know how to play bow arrow and pulled the arrow which targeted her eye. It started bleeding. While the parents returned home in the evening they gave a hot compress with hot rice. They thought it will heal (the injury). It got worse and painful later on. After a few days they took her to hospitals in Chitwan and Kathmandu. Yet she can’t see with that eye but it looks ok.”* (health volunteer #7)

### Perception about child injury prevention

Discussion on the measures of injury prevention was observed to be the most difficult part of all three focus groups. There were many pauses and silences. One of the mothers’ group participants observed *“what a difficult question?”* All three group participants mostly emphasised terms such as *“to be more careful”*, *“to be vigilant”*, *“to be watchful and alert”*, *“look after children” etc*. One thing that was most common among all participants was that people needed *“to be more careful while doing activities, such as driving, walking, riding a bicycle, playing or supervising children”.* They rarely mentioned concrete example of prevention of injuries. There was little recognition of “action oriented” preventive measures. A mother, whose son had fallen twice from the same balcony, had not put up any fencing or railings to protect the child. Instead, she had said -- *“now the child has grown up so there is no risk.”*

The health volunteers realised during the focus group discussion that they had little knowledge about the topic of injury prevention. However they sometimes had advised parents or family members about some injury prevention measures as a simple precaution to keep small children safe at home.

*“It’s not about injuries but we have told (mothers) to keep plastic bags away from children [from mouths of infants] in order to prevent them from suffocation… … but we haven’t thought whether it is relevant to our discussion.”* (health volunteer #7)

The level of knowledge and their efforts toward safety measures were found to be inadequate; consequently, action oriented measures of injury prevention were rarely observed. One of the mothers’ group said that as her children would soon be grown up, she would not need to worry about their injuries anymore! On the other hand, a grade seven student (boy) felt that injuries cannot be completely prevented.

*“Accidents cannot be eliminated however they can be reduced. Accidents do occur; it is not possible to eliminate them.”* (Student #6)

They also expressed a hope that injuries can be controlled or prevented by involving parents and community. The health volunteers believed that they could play an important role in preventing children from getting injuries, if they were provided with appropriate skills. Their opinion might have been influenced by their past experiences in addressing other child health issues.

In contrast, the students opined that such injuries are taking place because there are many factors responsible for such accidental injuries. They pointed out toward the responsibilities of parents, family members, teachers for the prevention of injuries. They also pointed out the role of injured person him/herself. Students also emphasised their [children’s] own responsibilities to stay away from injuries.

*“It is mainly the parents’ responsibility but not only the job of the parents; one should also remember one’s own duty [about staying safe].”* (student #9)

Some of the participants of student’s focus group felt that older ones should look after their younger siblings in order to keep them safe. While linking the student’s opinion about caring for younger siblings to the mother’s opinions, it was also expected by the mothers. The mothers of the focus group stated that other family members should also provide their time to care for children and their supervision, particularly when they are out to work.

*“Most of the mothers do not have time; other family members also don’t give time. All family members should cooperate, rather than leaving it all onto mothers. It should be considered as a job for other family members, including the father.”* (Mother #2, #9)

### Recognition of the problem of child injuries

All the health volunteer group members said that they became aware about issues related to child injuries and they felt that they would include some messages about injuries to children in regular meeting with mothers groups.

*“We have learnt many aspects of this topic now, we can tell every woman attending the women’s groups to be careful to prevent their children from injuries”* (all health volunteers unequivocally). *“Past is past. If we learn something now, about how to prevent injuries, we would be able to spread the knowledge and skill in the community.”* (health volunteer #7)

The mothers find it most difficult when this issue was raised for discussion as they did not have a particular example to mention. They were more vocal about restricting activities of children and their supervision. But as the discussion went on, they might have realised that something could have done. Mother participants of the focus group discussion said: *“We supervise them; look after them thinking whether the child may fall. We haven’t done anything else”* (mother #1 and #8).

At the later stage of the discussion, the mothers put forward some ideal examples on how to prevent injuries to children.

•Do not let them [children] walk at all.

•Ask someone to look after the child before going out for working

•The child below 5 years must not be left alone at all.

•All the members of family have to look after [the children]

•Railing should be placed at the same a strong (pukka) house is constructed.

•The back of the wooden ladders can be covered with wooden or tin plate.

•The fire must be lit only when it is required, extinguished after its use. [If there are] children at home, it must be completely extinguished after the use.

•Children should not be allowed to go closer to the kitchen or fireplace and a barrier can be placed around the fireplace.

•A door can be place in the kitchen and children should not be given with the hot [cooked] rice/meals [on their own].

A member of the student’s focus group explained how he contributed to his sibling’s safety at home. He mentioned that he had removed hazardous things from the reach of smaller children at his home. By doing so, he prevented them from burns and cut injuries. He said – *“We have kept matches and sharp instruments out of the reach of children”* (student #5).

In general, people do not have an idea of the measures of injury prevention; in our question on *“What we can do for our children so that the incidents of injuries are not repeated in future?”* most of the participants felt that children should be warned or taught/advised about the risks of injuries rather than giving examples such as separating risk factors from their environment.

One of the major issues that came from the mother’s focus group was about decision making at family level. In a typical Nepali family the father- or mother-in-laws decide about the role of the bride at home [22]. They are the one who send these women (mothers) to work indoors and, in the farms or outdoors. The mothers, although working outdoors, are blamed for not caring properly (by the in-laws) if their child gets injured or become unwell. On asking them, whether the grandparents do not look after these children, most of the mothers told they care of children only if they are doing well. This also points out towards equity at home.

### Prevention of child injuries

The Students mentioned about including injury prevention in school curricula and called for tougher traffic rule enforcement. They also stated about safety at schools.

The health volunteers were of an opinion that child injuries can also be prevented if children are provided with the necessary skills and knowledge. They also explained their contributions regarding health promotion in their communities as per the government policies. They were very keen to learn about injury prevention in order to educate the community in the context of changing pattern of illnesses.

*“We have already discussed how they got injured and broke their limbs as they start walking. I am wondering it would rather benefit from the discussion on how to educate parents [for prevention] than discussing about how or where the injuries occur… … How can we educate people about this? … … But there is no medicine for preventing falls from trees. [asking other participants] are there any treatments available? What can be given to them? There are no medicines which make the children not want to climb a tree? [laugh], similarly for children not wanting to jump!”* (health volunteer #7)

## Discussion

The qualitative study presented the findings from the opinions of 27 focus group participants from different parts of Makwanpur district. Overall a lack of knowledge about childhood injuries was observed. So child injuries were not a health priority and environmental risk factor modification were not identified by the group. Once participants were provided with some prompts, all of the participants were able to provide some examples of child injuries which had taken place in their communities and explain the incidents they knew about. The general perception about injuries was found to vary depending on the background experience of participants.

The participants expressed a lack of their familiarity with injuries during different points of discussion. Most of the participants, except some of the students, had diverse understanding about the definition of a child in contrast with the universal definition of a child (under the age of 18 years), as recommended by the Convention of the Rights of the Child (CRC). However, they identified that lack of supervision was a major risk factor for injuries among small children and also expressed their wish to contribute to injury prevention if provided with knowledge about how to do so. The opinions of participants covered a range of issues associated with child injuries.

It was agreed during the discussions that child injuries are neglected issues and were discussed formally for the first time in Makwanpur. There are no community or government activities aimed at preventing child injuries in Nepal. Child injury prevention initiatives need to encompass a broader range of activities from general awareness raising activities to specific ones. The need to address widespread traditional beliefs and superstitions related to child injuries was an important issue discussed.

Despite the frequent occurrence, child injuries are usually neglected and the awareness about their prevention is lacking in the communities. The most promising observation of this study is that community people were very keen to contribute in the prevention of injuries and to safeguarding their children. They were also keen on learning about ways to prevent their children from being injured. This may be the starting point of future endeavours on child injury prevention in Nepal.

Participants of the qualitative study identified different types and mechanisms of injuries: falls, road traffic injuries, burns, poisoning, drowning and animal related. However, road crashes and other vehicular injuries were found to be widely perceived types of injuries for most of the participants. People related injury causality with children’s behaviour, chance and surroundings which was also identified in a study conducted in Stockholm [26].

It has been found elsewhere that parents can identify the risk factors for child injuries and can describe preventive measures when solicited, but they need external input in terms of safeguarding children [13,27]. Participants of this study also suggested several measures for preventing or reducing child injuries. Training community residents on home visits, education, and risk reduction for child injury prevention was found to be helpful in low-income settings of South Africa [28,29]. The mobilisation of “Lay Health Workers” has shown promising benefit in reducing mortality and morbidity from common childhood illnesses by promoting immunisation, and breastfeeding; and also in improving TB treatment outcomes [30]. In Makwanpur, women groups have been successful at identifying and prioritising problems around perinatal health [31].

Injuries reflect social and personal environments of the injured person [32]. The concept of injury prevention is also varied between the different participants and depended upon their professional backgrounds as also described in the literature [6]. Qualitative studies on child injuries explore a range of factors elucidating their association with the occurrence of injury to children and its preventability: preventability of injuries at home [9], parents’ knowledge, attitude and beliefs related to child injuries [10,33], and knowledge and beliefs of mothers about child injuries [11]. The promising response of the female community volunteers in Makwanpur and their keenness to help in preventing injuries could be the starting point for developing a community-based child injury prevention programme.

Nevertheless, the opinions related to fatalism and superstitions in relation to the prevention of injuries were also heard from the participants. This could be a major challenge in establishing injury prevention programme because these beliefs are deeply rooted in the community.

### Strengths

The focus group discussions were conducted, alongside the household survey, in different locations with different groups of people from the local community. This resulted in gathering a broad range of information about childhood injuries and diverse opinions. These two approaches have made the information rich and helped validate the opinions.

In order to control the discussion and solicit the required information a checklist of issues was developed and used. The discussions were mainly focused around the knowledge and perception of injuries and practice of injury prevention activities. Full length discussions were recorded and detailed transcripts was prepared in both Nepali and English languages, which was very helpful to refer when the findings were written up.

The qualitative study also explored the existing infrastructure of keeping children safe from injuries and local professionals realised the need for this. The opinions of the focus group participants were generally positive and there was an interest in being involved in future community-based injury prevention and safety promotion activities. This study revealed community perspectives on occurrence, factors influencing childhood injuries, their treatment and prevention practices in the communities.

### Limitations

Only three FGDs were conducted because of time constraints; therefore the views of other groups of people such as the opinions of fathers and professionals were not exclusively incorporated. However, the opinions of key stakeholders were gathered using in-depth interviews of the key informants (not presented in this paper). This was also felt in the Makwanpur study because of including all types of injuries for discussion; an individual might be inclined to specific type of injury only according to their background, experience and interest. However, the researcher reminded the participants about other types of injuries during discussion; participants seemed to put their preferred examples.

Issues about understanding of terms and definitions of injuries might also have effect on the results. The understanding of the injury related terms was found to vary among the mothers, health volunteers, students and paramedics. The students’ focus group concentrated more on road traffic injuries than other injuries, perhaps due to lack of awareness of other injury types.

## Conclusion

This research work has attempted to explore the risk factors for injuries and the relationship with socio-economic status of Households. This is one of the first attempts to explore the problem of child injuries in Nepal. In the process of conducting the study, community people were enabled to think about the neglected problem of injuries among children which was widely appreciated by them. Thus, the study compiled a wide range of information which is anticipated to be helpful in conducting similar research elsewhere in the country and in developing a child injury prevention package in Nepal.

## Competing interests

The authors declare that they have no competing interests.

## Authors’ contributions

PRP prepared the draft of the article with the suggestions from ET. PP and ME provided critical comments on the first draft of the manuscript. DSM provided overall support from MIRA in carrying out the fieldwork in Nepal. All authors read approved the final manuscript.

## Pre-publication history

The pre-publication history for this paper can be accessed here:

http://www.biomedcentral.com/1471-2458/14/476/prepub
